# Advances in genetic variation in metabolism-related fatty liver disease

**DOI:** 10.3389/fgene.2023.1213916

**Published:** 2023-09-11

**Authors:** Fan Shi, Mei Zhao, Shudan Zheng, Lihong Zheng, Haiqiang Wang

**Affiliations:** ^1^ School of Heilongjiang University of Chinese Medicine, Harbin, China; ^2^ Department of Internal Medicine, Fourth Affiliated Hospital, Heilongjiang University of Chinese Medicine, Harbin, China; ^3^ Department of Internal Medicine, First Affiliated Hospital, Heilongjiang University of Chinese Medicine, Harbin, China

**Keywords:** MAFLD, genetic variation, PNPLA3, HSD17B13, therapeutic potential

## Abstract

Metabolism-related fatty liver disease (MAFLD) is the most common form of chronic liver disease in the world. Its pathogenesis is influenced by both environmental and genetic factors. With the upgrading of gene screening methods and the development of human genome project, whole genome scanning has been widely used to screen genes related to MAFLD, and more and more genetic variation factors related to MAFLD susceptibility have been discovered. There are genetic variants that are highly correlated with the occurrence and development of MAFLD, and there are genetic variants that are protective of MAFLD. These genetic variants affect the development of MAFLD by influencing lipid metabolism and insulin resistance. Therefore, in-depth analysis of different mechanisms of genetic variation and targeting of specific genetic variation genes may provide a new idea for the early prediction and diagnosis of diseases and individualized precision therapy, which may be a promising strategy for the treatment of MAFLD.

## 1 Introduction

Metabolic associated fatty liver disease (MAFLD) is a clinicopathological syndrome characterized by diffuse hepatocyte steatosis and lipid accumulation, formerly known as non-alcoholic fatty liver disease (NAFLD) (Kawaguchi et al., 2022). In early 2020, an international panel of 30 experts from 22 countries issued an international expert consensus statement on a new definition of MAFLD, proposing comprehensive and simple diagnostic criteria for MAFLD, and officially renamending NAFLD as MAFLD to highlight the causative link between fatty liver disease and metabolic dysfunction (Eslam et al., 2020). MAFLD can cause a range of diseases, including steatohepatitis, liver fibrosis, cirrhosis and eventually hepatocellular carcinoma, and has emerged as an important risk factor for liver failure and liver transplantation (Powell et al., 2021). MAFLD is a multi-systemic clinical disease that manifests itself not only in the liver but also has a wide range of extrahepatic manifestations, and findings have shown that patients with MAFLD are at increased risk for heart failure, obesity, type 2 diabetes mellitus, metabolic syndrome, chronic kidney disease, extrahepatic malignancies, cognitive impairment, polycystic ovary syndrome, osteoporosis, and hypothyroidism, among others (Kaya and Yilmaz, 2022; Pipitone et al., 2023; Wei et al., 2023). With changes in diet and lifestyle, the prevalence of MAFLD is increasing and it has gradually become the most common form of chronic liver disease worldwide, affecting more than one-third of the global population (Chan et al., 2022). Seriously endanger human health and cause huge economic burden to society (Eslam et al., 2020).

The pathogenesis of MAFLD is not fully understood. The widely accepted mechanism is the “two-strike” hypothesis, The first blow is liver lipid ectopic deposition and insulin resistance (IR), and the second blow is oxidative stress (Day and James, 1998). The liver is the central organ of lipid metabolism, secreting very low density lipoprotein (VLDL) on the one hand, and internalizing fatty acids and lipoproteins on the other (Heeren and Scheja, 2021), Liver is also the main target organ of insulin action, which plays a very important role in the control of blood glucose homeostasis. Hyperinsulinemia and IR are induced by increased levels of glucose and free fatty acids in the blood due to lack of exercise and a high sugar and fat diet. IR can reduce the intake of glucose by adipose tissue and skeletal muscle, induce hyperglycemia, promote the conversion of glucose into fatty acids (FAs) and triglycerides in hepatocytes, increase the new synthesis of liver fat, and lead to excess lipid deposition in the liver and fatty degeneration of the liver. However, the accumulation of lipid intermediates such as diglycerol ester (DAG) and ceramide in fatty liver leads to the inhibition of insulin signaling cascade, which induces the vicious cycle of IR and lipid deposition (Sakurai et al., 2021). Excessive accumulation of liver lipids can induce increased liver lipid toxicity and trigger cellular stress response, excessive elevation of oxidative stress level can lead to oxidative modification of specific DNA, protein and lipid metabolites, and thus cause cell damage. In addition, oxidative stress can also activate inflammatory bodies represented by NLRP3, release pro-inflammatory cytokines, induce hepatocyte necrosis, and promote the development of MAFLD (Clare et al., 2022).

The pathogenesis of MAFLD is complex, caused by an interaction between genetic, environmental, and metabolic disorders, The “two-strike” hypothesis is not a good explanation of the complexity of its pathogenesis, and has been gradually replaced by the “multiple strike” theory. The “multiple shocks” include genetic factors, insulin resistance, lipid metabolism disorders, hepatocyte steatosis, oxidative stress, inflammation, mitochondrial dysfunction, endoplasmic reticulum stress, epigenetic factors, intestinal flora disorders, etc. Multiple mechanisms work together to promote the occurrence and development of MAFLD (Bence and Birnbaum, 2021). With the continuous development of genetics, the genetic characteristics of MAFLD have been revealed to a large extent, and the role of genetic factors in the occurrence and development of MAFLD has been increasingly understood, which may involve intra-hepatic lipolysis, triglyceride output, hepatic mitochondrial oxidation or glucokinase activity (Sangro et al., 2023). Specific genetic risk variants have been shown to play a critical role in MAFLD and amplify the effect of MAFLD on disease outcome, increasing liver-related and overall mortality (Pingitore and Romeo, 2019).

## 2 Major genetic predisposition in MAFLD

MAFLD is a metabolic liver injury that is closely related to genetic susceptibility, and the clinical and phenotypic variation in patients may be caused by genetic variation factors (Dabravolski et al., 2021; Xia et al., 2021), and it is associated with a Single nucleotide polymorphism (SNP) in multiple related gene loci (Zhu et al., 2022). SNP refers to the DNA sequence polymorphism caused by the change of a single nucleotide at the genome level, which is mostly the conversion or reversal of a single base. With advances in high-throughput sequencing technology, genome-wide association Studies (GWAS) have provided insight into the heritage background of MAFLD (Sveinbjornsson et al., 2022). To identify the genetic variation associated with MAFLD, a study involving a large European population sample based on GWAS identified five genetic variation loci that may influence susceptibility to MAFLD, they were located near GCKR, TR1B1, TM6SF2, APOE and PNPLA3(Ghodsian et al., 2021). In addition, more and more genetic factors have been identified as genetic modification factors of MAFLD (Liao et al., 2022).

### 2.1 PNPLA3

**FIGURE 1:**
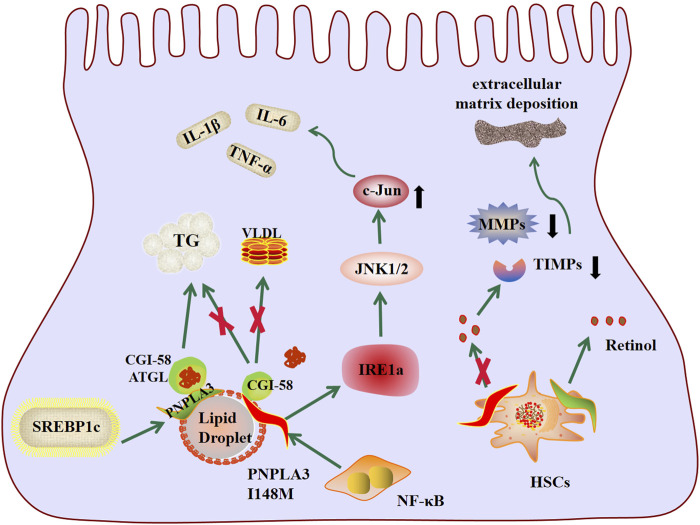
Schematic diagram of pathogenesis of genetic variation of PNPLA3 I148M.

PNPLA3 protein, an enzyme with lipase activity for triglycerides and retinol esters and acyltransferase activity for phospholipids, is a key regulator of lipid droplets in hepatocytes and hepatic stellate cells, mapping of human PNPLA3 gene to chromosome 22, it has 9 exons encoding 481 amino acids, belongs to the Patatin-like phospholipase family, and is the most highly expressed in human hepatocytes and hepatic stellate cells (Pingitore and Romeo, 2019). PNPLA3 has attracted great attention in the field of liver since Romeo et al. discovered a non-synonymous variant (rs738409, I148M) in PNPLA3 via GWAS that is significantly associated with liver fat content (Romeo et al., 2008). The I148M variant of PNPLA3 is a SNP caused by the substitution of isoleucine (I) with methionine (M) in amino acid coding sequence 148(I148M) (Xiang et al., 2021). The incidence of PNPLA3I148M varies in different populations, and studies have shown a major association between the PNPLA3I148M variant and MAFLD in women, but not in men (Chen et al., 2015). The incidence of PNPLA3I148M is higher in Hispanic populations than in European-American and African American populations (Romeo et al., 2008). PNPLA3 I148M is a genetic variant highly associated with the occurrence and development of MAFLD (Basu Ray, 2019; Salari et al., 2021). In order to elucidate the mechanism by which genes and their variants affect disease development, human induced pluripotent stem cells (hiPSC) have been used to establish a PNPLA3-related NAFLD model. This *in vitro* system provides a platform for elucidation of the complete pathophysiology of the PNPLA3I148M variant in NAFLD and drug development. While minimizing reliance on live animal models, the results show that I148M variants lead to increased lipid accumulation and susceptibility to hepatotoxins (Tilson et al., 2021). Previous studies have demonstrated that PNPLA3(148M) variation is associated with a variety of liver diseases, including MAFLD, non-alcoholic steatohepatitis (NASH), fibrosis, cirrhosis, and hepatocellular carcinoma (HCC) (Dhar and Loomba, 2021), and increases the risk of liver-related death (Grimaudo et al., 2020; Stender and Loomba, 2020). The genetic locus has been identified as having the most powerful role in increasing liver disease (Shang and Mashek, 2020). Despite the importance of PNPLA3I148M, the underlying pathogenic mechanism behind this variation remains unclear and may be related to lipid metabolism disorder, inflammation, and pro-fibrosis (Figure 1).

#### 2.1.1 Promote lipid accumulation

In hepatocytes, PNPLA3 is regulated by carbohydrate response element-binding protein (ChREBP) and sterol regulatory element-binding protein 1c (SREBP1c), has hydrolase activity on triglycerides, and plays an important role in lipid drome remodeling and very low density lipoprotein (VLDL) secretion in hepatocytes (BasuRay et al., 2019). I148M replacement results in loss of PNPLA3 lipase activity, impaired lipid catabolism, lipid droplet remodeling and VLDL secretion, VLDL secretion is the pathway by which fat escapes from the liver, thus leading to the accumulation of triglycerides in hepatocytes (Qadri et al., 2020; Akkiz et al., 2021). In addition, I148M is associated with decreased ubiquitination, resulting in accumulation of PNPLA3 in the liver and decreased TG mobilization in lipid droplet (LD) (BasuRay et al., 2017). The efficacy of CGI-58 as a co-activator of adipose triglyceride lipase (ATGL) in promoting intracellular fat decomposition has been well known. CGI-58 was able to combine with ATGL to promote fat decomposition and lipid phagocytosis, leading to LD degradation (Yu et al., 2020). PNPLA3I148M may inhibit ATGL catalyzed lipolysis by binding with CGI-58, leading to inhibition of liphagy, and thus increasing liver LD accumulation. The ability of PNPLA3 I148M to promote steatosis was weakened in mice lacking liver CGI-58 (Wang et al., 2019), further consolidating the key role of CGI-58 in mediating the action of PNPLA3 I148M.

#### 2.1.2 Promote inflammatory response

In addition to fat accumulation, inflammation is an important factor in the progression of MAFLD (Khanmohammadi and Kuchay, 2022). Overexpression of PNPLA3I148M leads to phosphorylation of STAT3 and activation of downstream inflammatory pathways, leading to the production of inflammatory substances such as IL-1β and IL-6, activates hepatic stellate cells, and further accelerates the process of NASH with fibrosis (Banini et al., 2021). NF-kB is the most important transcription factor that regulates inflammation and is involved in the progression of MAFLD. It was found that PNPLA3 I148M was activated by the transcription of NF-kB, and PNPLA3 I148M protein activated IRE1a signal of endoplasmic reticulum stress, then phosphorylated JNK1/2, upregulated the expression of c-Jun, and finally upregulated the expression of C-Jun-dependent inflammatory cytokines, such as TNF-α, and promote inflammation (Yuan et al., 2020). Enhanced IL-6/STAT3 signaling has been observed in PNPLA3I148M liver culture, which plays a central role in inflammation (Hirano, 2021), and can promote the progression of PNpla3I148M-induced MAFLD. IL-6 is a multipotent inflammatory cytokine involved in tissue homeostasis, regeneration and metabolism. IL-6 is significantly elevated in the liver of patients with MAFLD and correlates with severity, accelerating the progression of the disease, while blocking IL-6 signaling across the board reduces the progression of MAFLD (Park et al., 2023). Recruitment of these inflammatory cells eventually converges on hepatic stellate cells (HSCS), leading to activation.

#### 2.1.3 Regulation of hepatic stellate cells

Activation of hepatic stellate cells is a central factor in the progression of hepatic fibrosis. Upon liver injury, HSCs are activated, lose lipid-rich granules, and are transdifferentiated into a-smooth muscle actin (a-SMA)-positive myofibroblasts, which produce increased amount of ECM, proinflammatory, and profibrogenic cytokines, and cause liver fibrosis (Wang et al., 2022). In hepatic stellate cells, PNPLA3 is involved in retinol metabolism and has hydrolase activity on retinol ester, allowing the release of retinol from hepatic stellate cells. The hepatic stellate cells carrying PNPLA3I148M mutation lost the function of hydrolase activity, resulting in retinol intracellular retention. Impaired retinol production may lead to decreased secretion of matrix metalloproteinases and tissue inhibitors of metalloproteinases, resulting in extracellular matrix deposition and play an important role in hepatic stellate cell activation (Xiang et al., 2021). Human hepatic stellate cells with the PNPLA3(148M) variant showed higher expression of inflammatory cytokines and chemokines, and showed higher cell proliferation and migration (Bruschi et al., 2020). The genetic variation I148M has a significant effect on enhancing the pro-inflammatory response and pro-fibrosis characteristics of human hepatic stellate cells (Trepo et al., 2016; Bruschi et al., 2020). Thus, I148M variation in the PNPLA3 gene by regulating hepatic stellate cells is a risk factor for the development of severe hepatic fibrosis.

In conclusion, PNPLA3 is an important protein with a wide range of implications in metabolic liver diseases ranging from simple steatosis to cirrhosis and liver cancer, A better understanding of the biological function of PNPLA3 in lipid droplet metabolism will help advance the progress of disease treatment. This protein is an attractive target for the treatment of MAFLD, and targeting the PNPLA3(148M) variant is expected to be a promising direction for modern personalized medicine (Dong, 2019).

### 2.2 TM6SF2

Transmembrane 6 superfamily member 2 (TM6SF2) is located on chromosome 19 and encodes proteins containing 375 or 377 amino acids, respectively. The protein is expected to have 7–10 transmembrane domains, but does not contain any known functional domains and is mainly expressed in the gut and liver, suggesting a metabolism-related function (Zhang et al., 2018). TM6SF2 is localized in the endoplasmic reticulum (ER) and ER-Golgi intermediate region and plays a key role in intracellular lipidization of very low density lipoproteins, thereby preventing fatty liver disease (Luo et al., 2022b).

The non-synonymous variant of TM6SF2 (E167K, rs58542926) is characterized by cytosine (C) mutation of nucleotide 499 to thymine (T), and lysine-altered glutamic acid encoding codon 167 (E167K) (Jiang et al., 2021). The misfolding of the E167K variant protein accelerates protein degradation, resulting in decreased TM6SF2 protein levels and gene function. The loss of function leads to a decrease in the number of lipoprotein particles responsible for lipidation and lipoprotein, resulting in very low density lipoprotein (VLDL) retention in the liver and increased TAG content in the liver. Therefore, it is considered to be an important risk factor for lipid metabolism-related diseases (Li et al., 2018). Studies have shown that liver specific TM6SF2 loss impairs VLDL secretion, promotes hepatic steatosis and fibrosis, and accelerates HCC development (Newberry et al., 2021). Loss of TM6SF2 also strongly affects the structure of endoplasmic reticulum and mitochondria, thereby increasing ER stress and oxidative stress. Endoplasmic reticulum stress is closely related to lipogenesis or lipolysis, interfering with lipid metabolism and ultimately leading to inflammation and liver cell damage (Flessa et al., 2022), playing an important role in the progression of MAFLD (Longo et al., 2022). Clinical and epidemiological studies have confirmed the role of the TM6SF2 variant in the development of MAFLD (Xue et al., 2022), and TM6SF2 has also been shown to play an important role in promoting liver fibrosis and hepatocellular carcinoma in mouse models (Luo et al., 2022a).

### 2.3 GCKR

The Glucose kinase regulatory protein gene (GCKR) is located on human chromosome 2p23.3, approximately 27 kb in length, consisting of 19 exons and 18 introns. The GCKR gene encodes a protein consisting of 625 amino acids called Glucose kinase regulatory protein (GKRP), which is a member of the glucose isomerase family. GKRP is a heat-resistant chemical protein. Glucose kinase (GCK), a phosphorylase related to glucose metabolism, phosphorylates glucose to form glucose 6-phosphate, thus regulating liver glucose metabolism and promoting liver lipid generation. By regulating the activity of GCK, GKRP can further affect the utilization of glucose by liver cells, regulate body metabolism and resynthesis of fat (Peter et al., 2011). In hypoglycemia, GKRP binds to GCK to inactivate it and thus prevent GCK from entering the nucleus, reducing glucose decomposition and raising blood sugar; When blood sugar rises, GKRP separates from GCK, and the GCK in the nucleus restarts and returns to the cytoplasm, promoting glycolysis and lowering plasma glucose levels. Thus, GCKR encodes GKRP, which is involved in the regulation of glucose homeostasis and blood glucose control, thereby regulating glucose flow into hepatocytes and thereby inducing new adipogenesis (DNL) (Meroni et al., 2021). A SNP in GCKR, rs1260326 c.1337C > T p. P446L, is a proline to leucine substitution encoding the amino acid position of GCKR protein 446 (P446L). GCKR P446L is a functionally deficient variant that increases fat production by inducing glycolysis (Carlsson et al., 2020). The variation of GCKR is associated with a variety of lipid metabolism disorders (Fernandes Silva et al., 2019), significantly affected the MAFLD (Yuan et al., 2022). A meta-analysis showed that both the GCKR rs780094 and rs1260326 polymorphisms were significantly associated with an increased risk of MAFLD (Li et al., 2021).

### 2.4 APOE

The apolipoprotein E (Apo E) gene is located in the 2 band of the 13 region on the long arm of human chromosome 19, containing 4 exons and 3 introns (19q13.2). Apo E is a polymorphic protein synthesized mainly by the liver that influences lipid metabolism by binding to chylomicron and LDL receptors to mediate the clearance of very low density lipoproteins in serum (Zhou et al., 2020). The genetic variation of Apo E is consistently associated with increased liver fat content (Nascimento et al., 2020). The variant APOE gene obstructs the clearance of circulating lipoproteins and may hinder the reuptake of lipids in the liver, thus failing to timely transport excess cholesterol in the blood, resulting in lipid accumulation. These findings may help to better elucidate the genetic susceptibility associated with the onset and progression of MAFLD (Jamialahmadi et al., 2021).

### 2.5 TRIB1

The nerve cell death inducing protein kinase 1 (TRIB1) gene is located on human chromosome 8 and encodes the tribbles protein (Zhang et al., 2019), Although proteins of the TRIB1 family are pseudokinases that lack typical phosphotransferase activity, this pseudokinase plays a key role in normal and disease biology through its function as signal transduction mediators and protein scaffolds (Zhang et al., 2021). TRIB1 is closely related to lipid metabolism and downregulates liver lipogenesis through multi-molecular interactions (Ishizuka et al., 2014). Promotes ubiquitination of the transcription factor CCAAT enhancer binding protein α, thereby promoting its degradation (Quiroz-Figueroa et al., 2021). Downregulated ChREBP inhibits the expression of lipogenic genes and reduces the secretion of very low density lipoprotein, thus reducing the accumulation of lipids in the liver (Iwamoto et al., 2015). The TRIB1 rs17321515 variant has been identified by GWAS as a risk site for MAFLD (Vujkovic et al., 2022). In addition, studies have confirmed that TRIB1 rs17321515 gene polymorphism increases the risk of MAFLD patients in Han Chinese (Liu et al., 2019a; Liu et al., 2019b).

### 2.6 MBOAT7

The membrane bound O-acyltransferase 7 domain (MBOAT7), also known as lysophosphatidyl inositol acyltransferase 1 (LPIAT1), is a gene encoding acyltransferase activity and is located on human chromosome 19 (Sharma and Mandal, 2022). The acyltransferase activity encoding lysophosphatidyl inositol is involved in the conversion of fatty acids between phosphoesters and lysophosphoesters (Varadharajan et al., 2022). Experiments showed that the deficiency of MBOAT7 in mice remodeled PI and lysophosphatidylinositol (LPI) liver levels and promoted hyperinsulinemia and hepatic insulin resistance (Massey et al., 2023). MBOAT7 deficiency in mice and humans leads to fibrosis, suggesting that this may be an inflammation-independent hepatic fibrosis pathway mediated by lipid signaling (Thangapandi et al., 2021). rs641738C > T variation in the MBOAT7 gene caused damage to arachidonic acid phosphatidylinositol and promoted intracellular liver fat deposition, resulting in the conversion of saturated lysate phosphatidylinositol to triglycerides, thus promoting adipogenesis (Ismaiel and Dumitrascu, 2020). The study demonstrated that the rs641738C>T variant near MBOAT7 was a risk factor for the presence and severity of MAFLD in individuals of European ancestry (Teo et al., 2021).

### 2.7 HSD17B13

The human HSD17B13 gene is located on chromosome 4q22.1, with a total length of about 17kb, 8 exons and 7 introns (Chen et al., 2020). HSD17B13 belongs to the HSD17B family. HSD17B13 is the most highly expressed lipid droplet associated protein enriched in liver with hepatocellular specificity. HSD17B1 is selectively expressed in hepatocytes and localized only to the surface of lipid droplets. HSD17B13 also acts as a retinoic dehydrogenase that converts retinol to retinoic acid (RA), and its elevated level is closely related to the development of MAFLD (Zhang et al., 2021). A genetic variant of HSD17B13 (rs72613567, T > TA) was first described in 2018 and was associated with a reduced risk of MAFLD (Abul-Husn et al., 2018), other variants of HSD17B13 (rs6834314, A > G and rs9992651, G > A) were later associated with lower inflammatory scores in patients with MAFLD, which may be related to protection against MAFLD (Motomura et al., 2021). A meta-analysis showed that a polymorphism of the HSD17B13rs72613567:TA allele variant was associated with a reduced risk of HCC and MAFLD in the entire study population (Tang et al., 2021). Other studies have found that there may be interaction between HSD17B13 rs72613567 gene variation and PNPLA3 rs738409, which directly affect the expression level of PNPLA3 mRNA in liver and reduce the activity of PNPLA3 p. I148 (Abul-Husn et al., 2018). In a multiracial cohort of Asian patients with NAFLD, variants of HSD17B13 rs72613567 and rs6834314 were negatively associated with MAFLD and NASH and were associated with a lower incidence of adverse liver outcomes (Ting et al., 2021). Therefore, HSD17B13 is considered as a potential therapeutic target for MAFLD. However, it should be noted that the HSD17B13rs72613567 variant appears to be a risk variant for liver fibrosis in the Chinese Han MAFLD population, which is inconsistent with previous conclusions, indicating that future studies need to be verified by different ethnic groups, and it is necessary to study different groups in genetic studies. To map the genome-wide association study signal in detail (Liu et al., 2021).

### 2.8 Klotho

Klotho (KL) is a gene related to aging. It is located in 13q12. The total length of the 50 kb gene contains 4 introns and 5 exons, and has various biological effects such as antioxidant, anti-inflammatory and anti-apoptosis (Martin-Gonzalez et al., 2022). The human KL gene encodes alpha-Klotho protein, a multifunctional protein that regulates phosphate, calcium, and vitamin D metabolism, it is mainly expressed as a single transmembrane glycoprotein in kidney, parathyroid, brain and adipose tissue, and as a co-receptor of fibroblast growth factor 23(FGF23) (Kuro, 2019). A study using an animal model found that klotho knockout mice were extremely emaciated and significantly reduced fat accumulation in the liver, suggesting that it may play a direct role in the pathophysiology of fatty liver disease (Ohnishi et al., 2011). The KLrs495392 polymorphism was found to be protective against severe hepatic steatosis in patients with MAFLD and may reduce the risk of severe steatosis caused by the PNPLA3rs738409G variant (Liu et al., 2022). These data suggest that Klotho may be a therapeutic target for fatty liver disease and warrant further investigation (Chi et al., 2023).

In addition, there are many potential genetic modification factors. Klotho beta (KLB) is a protein-coding gene located on chromosome 4p14. The gene belongs to the glycoside hydrolase family 1 gene group. It is mainly expressed in fat and liver, KLB gene polymorphism is associated with obesity and liver inflammation, and may be involved in the pathogenesis of MAFLD (Ji et al., 2019). For example, the KLBrs17618244 gene variant was associated with liver damage in adult MAFLD patients, even more so in the presence of obesity stratification (Panera et al., 2021). An experiment based on MRI-PDFF and liver biopsy to study the clinical, laboratory, and genetic characteristics of MAFLD patients in A Chinese population found that patients with a variant allele of UQCC1 had low LFC, and UQCC1rs878639 (A>G) was identified as having a protective effect against MAFLD (Yang et al., 2022). Mitochondrial amidoxme reducing Component 1 (MARC1) variant rs2642438 increases phosphatidylcholine in human liver and reduces the severity of non-alcoholic fatty liver disease (Luukkonen et al., 2020).

**FIGURE 2:**
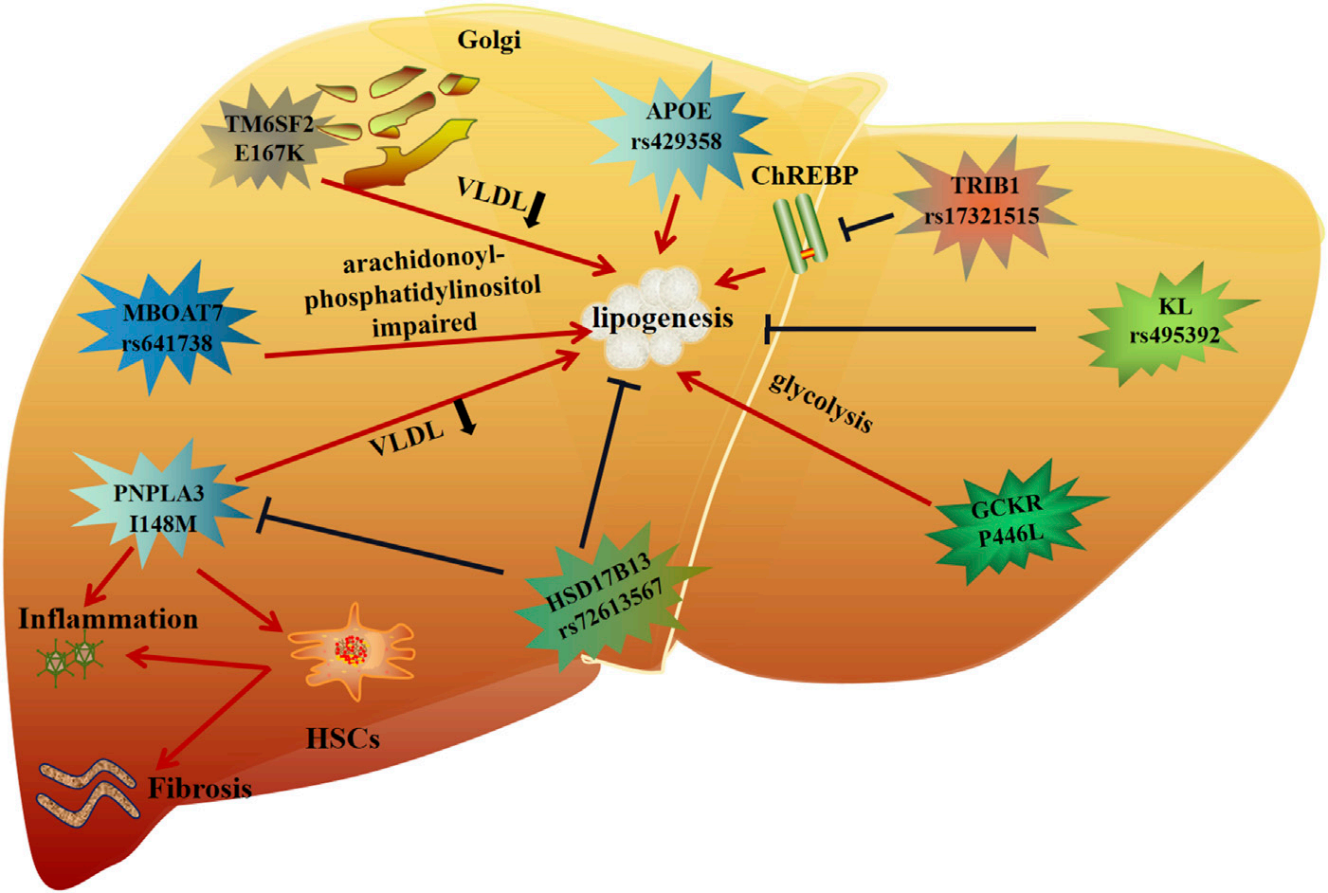
Molecular mechanism of genetic variation in MAFLD.

In summary, it can be seen that genetic polymorphism is closely related to lipid metabolism, inflammation and fibrosis in MAFLD (Figure 2). These genetic variants are either risk factors or protective factors. Therefore, in-depth analysis of genetic variants can improve our understanding of the pathogenesis of the disease, and may provide new targets and more personalized treatment for future MAFLD treatment.

## 3 Therapeutic potential associated with genetic variation factors

Currently, lifestyle interventions and weight loss remain the cornerstones of treatment for MAFLD (Stefano et al., 2023). Given the multifaceted pathophysiology of the disease, combination therapy such as lipid lowering, blood pressure lowering, glucose lowering, anti-obesity, antioxidant, anti-inflammatory and anti-fibrosis drugs may be considered a reasonable alternative (Makri et al., 2022). Some new signaling pathways and pharmacological targets are also being studied, such as cell therapy and intestinal flora regulation therapy (Raza et al., 2021), but no drug has been approved for the treatment of this disease alone. Genetic factors have been shown to be closely related to the onset and progression of MAFLD, suggesting that the exploration of genetic variants with diagnostic and therapeutic potential may have a promising clinical application prospect in the treatment of MAFLD (Romeo et al., 2020). Currently, the identification of people at risk for MAFLD patients is not accurate enough, and biomarkers to predict disease risk and treatment response are still lacking (Friedman et al., 2018). So on the one hand we can use these genetic factors to predict disease risk; on the other hand, therapeutic drugs can be developed by targeting genes that are particularly important.

### 3.1 Genetic risk prediction and early diagnosis

Liver biopsy is the gold standard diagnostic method for MAFLD, but it is limited due to its high cost and the possibility of complications due to the invasive procedure (Tsai and Lee, 2018). Genetic methods, especially SNPs, have received increasing attention in recent years due to their non-invasive applications, which combine the effects of a single SNP into a single score for predicting the risk of MAFLD. An increasing number of studies have demonstrated the utility of genetic risk scores (GRS) (June 2021). The higher the GRS, the higher the risk of liver–related disease MAFLD (Wang et al., 2021). GRS tools can be used to screen large numbers of people and, for those at high risk, can be prevented through aggressive lifestyle changes, increased physical activity and dietary modifications. For older adults who already have some degree of liver disease, these relevant genomic features may also guide more rigorous monitoring of liver disease complications (cirrhosis, liver cancer). It has been shown that inclusion of PNPLA3, TM6SF2 and HSD17B13 as genetic risk factors in a risk stratification model may improve its prediction of MAFLD severity and advanced fibrosis (Paternostro et al., 2021). Especially for children and adolescents, this population has relatively few other risk factors, so early predictive diagnosis with genetic biomarkers and early lifestyle interventions for those at risk may benefit patients the most (Li et al., 2020). The GRS tool can provide recommendations for lifestyle changes in genetically predisposed patients, but the effects on liver-related mortality and liver cancer development need to be determined in long-term studies (June 2021). Polygenic Risk Scores (PRS) can be used to predict MAFLDs non-invasively. For the increasing incidence of MAFLD-associated HCC, it is also necessary to develop reliable PRS for in-depth understanding of the causal relationship between NAFLD and HCC and to improve HCC risk stratification (Bianco et al., 2021). In the future, robust prediction models should also integrate rare and common variations and other risk factors, requiring very large samples to be developed (Dessein, 2021).

### 3.2 Development of potential therapeutic targets

Genetic variation plays an important role in the pathogenesis of MAFLD and its evolution into cirrhosis and HCC, so targeting the locus of genetic variation may be a promising therapeutic approach. Among them, antisense oligonucleotides (ASOs) are a novel therapeutic approach that targets homologous mRNA sequences, modulates gene expression or translation of related proteins, and ASOs can be easily injected under the skin and directly target mRNA molecules, resulting in faster and longer-lasting responses than directly inhibiting protein production (Scharner and Aznarez, 2021). Some studies have found that targeting PNPLA3I148M by ASOs at the RNA level may provide significant advantages for realizing long-term inhibition of the expression of risk variants in carriers (Cherubini et al., 2021). Liver targeting of triantennal n-acetyl galactosamine-coupled ASO mediated Pnpla3 silencing reduced liver lipogenesis and steatosis in mice carrying a human I148M mutant (Linden et al., 2019). More recently, ION839 (AZD2693), a PNpla3-targeting ASO, was used in patients with NASH and PNPLA3 I148M in a Phase I clinical trial (NCT04483947) (Xu et al., 2022). Although ASOs is a promising precision medicine drug, mild to moderate toxicity as well as some pro-inflammatory and platelet reduction manifestations can still be observed at long-term high doses, requiring further research (Goyenvalle et al., 2023). In addition to targeting PNPLA3 gene variants, liver-specific acetyl-CoA carboxylase (ACC) inhibitors (MK4074) block enhanced fatty acid synthesis, improve fatty acid beta-oxidation, and then reverse the NAFLD phenotype caused by TM6SF2 defects (Li et al., 2022). The protective HSD17B13 variant is also a potential therapeutic target, reproterol, a potential regulator of 17β-HSD13, was found to prevent MAFLD through phosphorylation of 17β-HSD13Ser33 mediated by PKA, so targeting the Ser33 phosphorylation site may be a potential treatment approach (Su et al., 2022).

## 3 Conclusion

Metabolically related fatty liver is a common chronic disease, but its pathogenesis remains unclear. Most current studies believe that it is the result of multiple factors such as genetic variation and environment. With the increasing prevalence of MAFLD worldwide, it is imperative to explore new and effective treatments. In the past decade, more and more genetic variants such as GCKR, TRIB1, TM6SF2, APOE, PNPLA3, HSD17B13, etc. have been discovered, which regulate the susceptibility and progression of MAFLD by affecting lipid metabolism, inflammatory response, insulin resistance, oxidative stress, liver fibrosis and other processes. Therefore, targeting specific genetic variants may provide new ideas for the early prediction and diagnosis of MAFLD and individualized precision therapy. For genes involved in the pathogenesis of MAFLD, drugs that silence or turn off the gene can be used to reduce the effect of the gene variant; for protective gene variants, the effect of protective mutations can be simulated to prevent MAFLD. However, it is important to note that further research is needed to expand on the molecular mechanisms associated with these new findings, for example, according to the current study, PNPLA3I148M is mainly involved in lipid accumulation in the liver, but the specific mechanisms need further research to clarify and further validate these results. All new therapies need to be extensively evaluated for safety and efficacy in clinical trials. To sum up, this is a very promising research area. Although still in its infancy, this field may open up a whole new avenue for the treatment of MAFLD, and there are many more potential therapeutic targets waiting to be explored and discovered in the future, giving us hope to conquer MAFLD in the future.

## References

[B1] Abul-Husn N. S. Cheng X. Li A. H. Xin Y. Schurmann C. Stevis P. (2018). A protein-truncating HSD17B13 variant and protection from chronic liver disease. N. Engl. J. Med. 378, 1096–1106. 10.1056/NEJMoa1712191 29562163 PMC6668033

[B2] Akkiz H. Taskin E. Karaogullarindan U. Delik A. Kuran S. andKutlu O. (2021). The influence of RS738409 I148M polymorphism of patatin-like phospholipase domain containing 3 gene on the susceptibility of non-alcoholic fatty liver disease. Med. Baltim. 100, e25893. 10.1097/MD.0000000000025893 PMC8133255 34106646

[B3] Banini B. A. Kumar D. P. Cazanave S. Seneshaw M. Mirshahi F. Santhekadur P. K. (2021). Identification of a metabolic, transcriptomic, and molecular signature of patatin-like phospholipase domain containing 3-mediated acceleration of steatohepatitis. Hepatol. Baltim. Md 73, 1290–1306. 10.1002/hep.31609 PMC8046714 33131062

[B4] Basu Ray S. (2019). PNPLA3-I148M: a problem of plenty in non-alcoholic fatty liver disease. Adipocyte 8, 201–208. 10.1080/21623945.2019.1607423 31062641 PMC6768214

[B5] Basuray S. Smagris E. Cohen J. C. andHobbs H. H. (2017). The PNPLA3 variant associated with fatty liver disease (I148M) accumulates on lipid droplets by evading ubiquitylation. Hepatology 66, 1111–1124. 10.1002/hep.29273 28520213 PMC5605398

[B6] Basuray S. Wang Y. Smagris E. Cohen J. C. andHobbs H. H. (2019). Accumulation of PNPLA3 on lipid droplets is the basis of associated hepatic steatosis. Proc. Natl. Acad. Sci. U. S. A. 116, 9521–9526. 10.1073/pnas.1901974116 31019090 PMC6511016

[B7] Bence K. K. Birnbaum M. J. (2021). Metabolic drivers of non-alcoholic fatty liver disease. Mol. Metab. 50, 101143. 10.1016/j.molmet.2020.101143 33346069 PMC8324696

[B8] Bianco C. Jamialahmadi O. Pelusi S. Baselli G. Dongiovanni P. Zanoni I. (2021). Non-invasive stratification of hepatocellular carcinoma risk in non-alcoholic fatty liver using polygenic risk scores. J. Hepatol. 74, 775–782. 10.1016/j.jhep.2020.11.024 33248170 PMC7987554

[B9] Bruschi F. V. Tardelli M. Einwallner E. Claudel T. andTrauner M. (2020). PNPLA3 I148M up-regulates hedgehog and yap signaling in human hepatic stellate cells. Int. J. Mol. Sci. 21, 8711. 10.3390/ijms21228711 33218077 PMC7698885

[B10] Carlsson B. Linden D. Brolen G. Liljeblad M. Bjursell M. Romeo S. (2020). Review article: the emerging role of genetics in precision medicine for patients with non-alcoholic steatohepatitis. Aliment. Pharmacol. Ther. 51, 1305–1320. 10.1111/apt.15738 32383295 PMC7318322

[B11] Chan K. E. Koh T. J. L. Tang A. S. P. Quek J. Yong J. N. Tay P. (2022). Global prevalence and clinical characteristics of metabolic-associated fatty liver disease: A meta-analysis and systematic review of 10 739 607 individuals. J. Clin. Endocrinol. Metab. 107, 2691–2700. 10.1210/clinem/dgac321 35587339

[B12] Chen H. Zhang Y. Guo T. Yang F. Mao Y. Li L. (2020). Genetic variant rs72613567 ofHSD17B13gene reduces alcohol‐related liver disease risk in Chinese Han population. Liver Int. 40, 2194–2202. 10.1111/liv.14616 33151633 PMC7496237

[B13] Chen L.-Z. Xin Y.-N. Geng N. Jiang M. Zhang D.-D. andXuan S.-Y. (2015). PNPLA3 I148M variant in nonalcoholic fatty liver disease: demographic and ethnic characteristics and the role of the variant in nonalcoholic fatty liver fibrosis. World J. Gastroenterology 21, 794–802. 10.3748/wjg.v21.i3.794 PMC4299331 25624712

[B14] Cherubini A. Casirati E. Tomasi M. andValenti L. (2021). PNPLA3 as a therapeutic target for fatty liver disease: the evidence to date. Expert Opin. Ther. Targets 25, 1033–1043. 10.1080/14728222.2021.2018418 34904923

[B15] Chi Z. Teng Y. Liu Y. Gao L. Yang J. andZhang Z. (2023). Association between klotho and non-alcoholic fatty liver disease and liver fibrosis based on the NHANES 2007–2016. Ann. Hepatology 28, 101125. 10.1016/j.aohep.2023.101125 37286168

[B16] Clare K. Dillon J. F. andBrennan P. N. (2022). Reactive oxygen species and oxidative stress in the pathogenesis of MAFLD. J. Clin. Transl. Hepatol. 10, 939–946. 10.14218/JCTH.2022.00067 36304513 PMC9547261

[B17] Dabravolski S. A. Bezsonov E. E. Baig M. S. Popkova T. V. Nedosugova L. V. Starodubova A. V. (2021). Mitochondrial mutations and genetic factors determining NAFLD risk. Int. J. Mol. Sci. 22, 4459. 10.3390/ijms22094459 33923295 PMC8123173

[B18] Day C. P. James O. F. (1998). Steatohepatitis: a tale of two "hits. Gastroenterology 114, 842–845. 10.1016/s0016-5085(98)70599-2 9547102

[B19] Dessein A. (2021). Clinical utility of polygenic risk scores for predicting NAFLD disorders. J. Hepatol. 74, 769–770. 10.1016/j.jhep.2021.02.005 33653592

[B20] Dhar D. Loomba R. (2021). Emerging metabolic and transcriptomic signature of PNPLA3-associated NASH. Hepatology 73, 1248–1250. 10.1002/hep.31735 33544416 PMC9683537

[B21] Dong X. C. (2019). PNPLA3-A potential therapeutic target for personalized treatment of chronic liver disease. Front. Med. (Lausanne) 6, 304. 10.3389/fmed.2019.00304 31921875 PMC6927947

[B22] Eslam M. Newsome P. N. Sarin S. K. Anstee Q. M. Targher G. Romero-Gomez M. (2020). A new definition for metabolic dysfunction-associated fatty liver disease: an international expert consensus statement. J. Hepatology 73, 202–209. 10.1016/j.jhep.2020.03.039 32278004

[B23] Eslam M. Sanyal A. J. George J. andInternational Consensus P. (2020). MAFLD: A consensus-driven proposed nomenclature for metabolic associated fatty liver disease. Gastroenterology 158, 1999–2014.e1. 10.1053/j.gastro.2019.11.312 32044314

[B24] Fernandes Silva L. Vangipurapu J. Kuulasmaa T. andLaakso M. (2019). An intronic variant in the GCKR gene is associated with multiple lipids. Sci. Rep. 9, 10240. 10.1038/s41598-019-46750-3 31308433 PMC6629684

[B25] Flessa C.-M. Kyrou I. Nasiri-Ansari N. Kaltsas G. Kassi E. andRandeva H. S. (2022). Endoplasmic reticulum stress in nonalcoholic (metabolic associated) fatty liver disease (NAFLD/MAFLD). J. Cell Biochem. 123, 1585–1606. 10.1002/jcb.30247 35490371

[B26] Friedman S. L. Neuschwander-Tetri B. A. Rinella M. andSanyal A. J. (2018). Mechanisms of NAFLD development and therapeutic strategies. Nat. Med. 24, 908–922. 10.1038/s41591-018-0104-9 29967350 PMC6553468

[B27] Ghodsian N. Abner E. Emdin C. A. Gobeil E. Taba N. Haas M. E. (2021). Electronic health record-based genome-wide meta-analysis provides insights on the genetic architecture of non-alcoholic fatty liver disease. Cell Rep. Med. 2, 100437. 10.1016/j.xcrm.2021.100437 34841290 PMC8606899

[B28] Goyenvalle A. Jimenez-Mallebrera C. Van Roon W. Sewing S. Krieg A. M. Arechavala-Gomeza V. (2023). Considerations in the preclinical assessment of the safety of antisense oligonucleotides. Nucleic Acid. Ther. 33, 1–16. 10.1089/nat.2022.0061 36579950 PMC9940817

[B29] Grimaudo S. Pipitone R. M. Pennisi G. Celsa C. Camma C. Di Marco V. (2020). Association between PNPLA3 rs738409 C>G variant and liver-related outcomes in patients with nonalcoholic fatty liver disease. Clin. Gastroenterol. Hepatol. 18, 935–944. 10.1016/j.cgh.2019.08.011 31419571

[B30] Heeren J. Scheja L. (2021). Metabolic-associated fatty liver disease and lipoprotein metabolism. Mol. Metab. 50, 101238. 10.1016/j.molmet.2021.101238 33892169 PMC8324684

[B31] Hirano T. (2021). IL-6 in inflammation, autoimmunity and cancer. Int. Immunol. 33, 127–148. 10.1093/intimm/dxaa078 33337480 PMC7799025

[B32] Ishizuka Y. Nakayama K. Ogawa A. Makishima S. Boonvisut S. Hirao A. (2014). TRIB1 downregulates hepatic lipogenesis and glycogenesis via multiple molecular interactions. J. Mol. Endocrinol. 52, 145–158. 10.1530/JME-13-0243 24389359

[B33] Ismaiel A. Dumitrascu D. L. (2020). Genetic predisposition in metabolic-dysfunction-associated fatty liver disease and cardiovascular outcomes—systematic review. Eur. J. Clin. Investigation 50, e13331. 10.1111/eci.13331 32589269

[B34] Iwamoto S. Boonvisut S. Makishima S. Ishizuka Y. Watanabe K. andNakayama K. (2015). The role of TRIB1 in lipid metabolism; from genetics to pathways. Biochem. Soc. Trans. 43, 1063–1068. 10.1042/BST20150094 26517924

[B35] Jamialahmadi O. Mancina R. M. Ciociola E. Tavaglione F. Luukkonen P. K. Baselli G. (2021). Exome-wide association study on alanine aminotransferase identifies sequence variants in the GPAM and APOE associated with fatty liver disease. Gastroenterology 160, 1634–1646.e7. 10.1053/j.gastro.2020.12.023 33347879

[B36] Ji F. Liu Y. Hao J.-G. Wang L.-P. Dai M.-J. Shen G.-F. (2019). KLB gene polymorphism is associated with obesity and non-alcoholic fatty liver disease in the Han Chinese. Aging (Albany NY) 11, 7847–7858. 10.18632/aging.102293 31548436 PMC6781984

[B37] Jiang X. Qian H. andDing W. X. (2021). New glance at the role of TM6SF2 in lipid metabolism and liver cancer. Hepatology 74, 1141–1144. 10.1002/hep.31851 33826777

[B38] Jun D. W. (2021). An analysis of polygenic risk scores for non-alcoholic fatty liver disease. Clin. Mol. Hepatol. 27, 446–447. 10.3350/cmh.2021.0133 34024056 PMC8273633

[B39] Kawaguchi T. Tsutsumi T. Nakano D. andTorimura T. (2022). MAFLD: renovation of clinical practice and disease awareness of fatty liver. Hepatol. Res. 52, 422–432. 10.1111/hepr.13706 34472683

[B40] Kaya E. Yilmaz Y. (2022). Metabolic-associated fatty liver disease (MAFLD): A multi-systemic disease beyond the liver. J. Clin. Transl. Hepatol. 10, 329–338. 10.14218/JCTH.2021.00178 35528971 PMC9039705

[B41] Khanmohammadi S. Kuchay M. S. (2022). Toll-like receptors and metabolic (dysfunction)-associated fatty liver disease. Pharmacol. Res. 185, 106507. 10.1016/j.phrs.2022.106507 36252773

[B42] Kuro O. M. (2019). The Klotho proteins in health and disease. Nat. Rev. Nephrol. 15, 27–44. 10.1038/s41581-018-0078-3 30455427

[B43] Li J. Hua W. Ji C. Rui J. Zhao Y. Xie C. (2020). Effect of the patatin-like phospholipase domain containing 3 gene (PNPLA3) I148M polymorphism on the risk and severity of nonalcoholic fatty liver disease and metabolic syndromes: A meta-analysis of paediatric and adolescent individuals. Pediatr. Obes. 15, e12615. 10.1111/ijpo.12615 32020770

[B44] Li J. Zhao Y. Zhang H. Hua W. Jiao W. Du X. (2021). Contribution of Rs780094 and Rs1260326 polymorphisms in GCKR gene to non-alcoholic fatty liver disease: A meta-analysis involving 26,552 participants. Endocr. Metab. Immune Disord. Drug Targets 21, 1696–1708. 10.2174/1871530320999201126202706 33243135

[B45] Li T. T. Li T. H. Peng J. He B. Liu L. S. Wei D. H. (2018). TM6SF2: A novel target for plasma lipid regulation. Atherosclerosis 268, 170–176. 10.1016/j.atherosclerosis.2017.11.033 29232562

[B46] Liu W. Y. Zhang X. Li G. Tang L. J. Zhu P. W. Rios R. S. (2022). Protective association of Klotho rs495392 gene polymorphism against hepatic steatosis in non-alcoholic fatty liver disease patients. Clin. Mol. Hepatol. 28, 183–195. 10.3350/cmh.2021.0301 34839623 PMC9013609

[B47] Li Z. Y. Wu G. Qiu C. Zhou Z. J. Wang Y. P. Song G. H. (2022). Mechanism and therapeutic strategy of hepatic TM6SF2-deficient non-alcoholic fatty liver diseases via *in vivo* and *in vitro* experiments. World J. Gastroenterol. 28, 2937–2954. 10.3748/wjg.v28.i25.2937 35978872 PMC9280743

[B48] Liao S. An K. Liu Z. He H. An Z. Su Q. (2022). Genetic variants associated with metabolic dysfunction-associated fatty liver disease in western China. J. Clin. Lab. Anal. 36, e24626. 10.1002/jcla.24626 35881683 PMC9459258

[B49] Linden D. Ahnmark A. Pingitore P. Ciociola E. Ahlstedt I. Andreasson A. C. (2019). Pnpla3 silencing with antisense oligonucleotides ameliorates nonalcoholic steatohepatitis and fibrosis in Pnpla3 I148M knock-in mice. Mol. Metab. 22, 49–61. 10.1016/j.molmet.2019.01.013 30772256 PMC6437635

[B50] Liu Q. Liu S. S. Zhao Z. Z. Zhao B. T. Du S. X. Jin W. W. (2019a). TRIB1 rs17321515 gene polymorphism increases the risk of coronary heart disease in general population and non-alcoholic fatty liver disease patients in Chinese Han population. Lipids Health Dis. 18, 165. 10.1186/s12944-019-1108-2 31470861 PMC6717352

[B51] Liu Q. Xue F. Meng J. Liu S. S. Chen L. Z. Gao H. (2019b). TRIB1 rs17321515 and rs2954029 gene polymorphisms increase the risk of non-alcoholic fatty liver disease in Chinese Han population. Lipids Health Dis. 18, 61. 10.1186/s12944-019-1001-z 30851741 PMC6408849

[B52] Liu W. Y. Eslam M. Zheng K. I. Ma H. L. Rios R. S. Lv M. Z. (2021). Associations of hydroxysteroid 17-beta dehydrogenase 13 variants with liver histology in Chinese patients with metabolic-associated fatty liver disease. J. Clin. Transl. Hepatol. 9, 194–202. 10.14218/JCTH.2020.00151 34007801 PMC8111109

[B53] Longo M. Meroni M. Paolini E. Erconi V. Carli F. Fortunato F. (2022). TM6SF2/PNPLA3/MBOAT7 loss-of-function genetic variants impact on NAFLD development and progression both in patients and in *in vitro* models. Cell Mol. Gastroenterol. Hepatol. 13, 759–788. 10.1016/j.jcmgh.2021.11.007 34823063 PMC8783129

[B54] Luo F. Oldoni F. andDas A. (2022a). TM6SF2: A novel genetic player in nonalcoholic fatty liver and cardiovascular disease. Hepatol. Commun. 6, 448–460. 10.1002/hep4.1822 34532996 PMC8870032

[B55] Luo F. Smagris E. Martin S. A. Vale G. Mcdonald J. G. Fletcher J. A. (2022b). Hepatic TM6SF2 is required for lipidation of VLDL in a pre-golgi compartment in mice and rats. Cell Mol. Gastroenterol. Hepatol. 13, 879–899. 10.1016/j.jcmgh.2021.12.008 34923175 PMC8804273

[B56] Luukkonen P. K. Juuti A. Sammalkorpi H. Penttila A. K. Oresic M. Hyotylainen T. (2020). MARC1 variant rs2642438 increases hepatic phosphatidylcholines and decreases severity of non-alcoholic fatty liver disease in humans. J. Hepatol. 73, 725–726. 10.1016/j.jhep.2020.04.021 32471727

[B57] Makri E. S. Makri E. andPolyzos S. A. (2022). Combination therapies for nonalcoholic fatty liver disease. J. Pers. Med. 12, 1166. 10.3390/jpm12071166 35887662 PMC9322793

[B58] Martin-Gonzalez C. Espelosin-Ortega E. Abreu-Gonzalez P. Fernandez-Rodriguez C. Vera-Delgado V. E. Gonzalez-Navarrete L. (2022). Klotho levels and their relationship with inflammation and survival among alcoholic patients. Biomolecules 12, 1151. 10.3390/biom12081151 36009045 PMC9405938

[B59] Massey W. J. Varadharajan V. Banerjee R. Brown A. L. Horak A. J. Hohe R. C. (2023). MBOAT7-driven lysophosphatidylinositol acylation in adipocytes contributes to systemic glucose homeostasis. J. Lipid Res. 64, 100349. 10.1016/j.jlr.2023.100349 36806709 PMC10041558

[B60] Meroni M. Longo M. Tria G. andDongiovanni P. (2021). Genetics is of the essence to face NAFLD. Biomedicines 9, 1359. 10.3390/biomedicines9101359 34680476 PMC8533437

[B61] Motomura T. Amirneni S. Diaz-Aragon R. Faccioli L. Malizio M. Coard M. (2021). Is HSD17B13 genetic variant a protector for liver dysfunction? Future perspective as a potential therapeutic target. J. Personalized Med. 11, 619. 10.3390/jpm11070619 PMC8304981 34208839

[B62] Nascimento J. C. R. Matos G. A. Pereira L. C. Mourao A. Sampaio A. M. Oria R. B. (2020). Impact of apolipoprotein E genetic polymorphisms on liver disease: an essential review. Ann. Hepatol. 19, 24–30. 10.1016/j.aohep.2019.07.011 31548169

[B63] Newberry E. P. Hall Z. Xie Y. Molitor E. A. Bayguinov P. O. Strout G. W. (2021). Liver-specific deletion of mouse Tm6sf2 promotes steatosis, fibrosis, and hepatocellular cancer. Hepatology 74, 1203–1219. 10.1002/hep.31771 33638902 PMC8390580

[B64] Ohnishi M. Kato S. Akiyoshi J. Atfi A. andRazzaque M. S. (2011). Dietary and genetic evidence for enhancing glucose metabolism and reducing obesity by inhibiting klotho functions. FASEB J. 25, 2031–2039. 10.1096/fj.10-167056 21382979 PMC3101030

[B65] Panera N. Meroni M. Longo M. Crudele A. Valenti L. Bellacchio E. (2021). The KLB rs17618244 gene variant is associated with fibrosing MAFLD by promoting hepatic stellate cell activation. EBioMedicine 65, 103249. 10.1016/j.ebiom.2021.103249 33640795 PMC7921469

[B66] Park J. Zhao Y. Zhang F. Zhang S. Kwong A. C. Zhang Y. (2023). IL-6/STAT3 axis dictates the PNPLA3-mediated susceptibility to non-alcoholic fatty liver disease. J. Hepatol. 78, 45–56. 10.1016/j.jhep.2022.08.022 36049612 PMC9772150

[B67] Paternostro R. Staufer K. Traussnigg S. Stattermayer A. F. Halilbasic E. Keritam O. (2021). Combined effects of PNPLA3, TM6SF2 and HSD17B13 variants on severity of biopsy-proven non-alcoholic fatty liver disease. Hepatol. Int. 15, 922–933. 10.1007/s12072-021-10200-y 34076851 PMC8382644

[B68] Peter A. Stefan N. Cegan A. Walenta M. Wagner S. Königsrainer A. (2011). Hepatic glucokinase expression is associated with lipogenesis and fatty liver in humans. J. Clin. Endocrinol. Metabolism 96, E1126–E1130. 10.1210/jc.2010-2017 21490074

[B69] Pingitore P. Romeo S. (2019). The role of PNPLA3 in health and disease. Biochim. Biophys. Acta Mol. Cell Biol. Lipids 1864, 900–906. 10.1016/j.bbalip.2018.06.018 29935383

[B70] Pipitone R. M. Ciccioli C. Infantino G. La Mantia C. Parisi S. Tulone A. (2023). Mafld: a multisystem disease. Ther. Adv. Endocrinol. Metab. 14, 20420188221145549. 10.1177/20420188221145549 36726391 PMC9885036

[B71] Powell E. E. Wong V. W. andRinella M. (2021). Non-alcoholic fatty liver disease. Lancet 397, 2212–2224. 10.1016/S0140-6736(20)32511-3 33894145

[B72] Qadri S. Lallukka-Bruck S. Luukkonen P. K. Zhou Y. Gastaldelli A. Orho-Melander M. (2020). The PNPLA3-I148M variant increases polyunsaturated triglycerides in human adipose tissue. Liver Int. 40, 2128–2138. 10.1111/liv.14507 32386450

[B73] Quiroz-Figueroa K. Vitali C. Conlon D. M. Millar J. S. Tobias J. W. Bauer R. C. (2021). TRIB1 regulates LDL metabolism through CEBPα-mediated effects on the LDL receptor in hepatocytes. J. Clin. Invest. 131, e146775. 10.1172/JCI146775 34779419 PMC8592541

[B74] Raza S. Rajak S. Upadhyay A. Tewari A. andAnthony Sinha R. (2021). Current treatment paradigms and emerging therapies for NAFLD/NASH. Front. Biosci. (Landmark Ed. 26, 206–237. 10.2741/4892 33049668 PMC7116261

[B75] Romeo S. Kozlitina J. Xing C. Pertsemlidis A. Cox D. Pennacchio L. A. (2008). Genetic variation in PNPLA3 confers susceptibility to nonalcoholic fatty liver disease. Nat. Genet. 40, 1461–1465. 10.1038/ng.257 18820647 PMC2597056

[B76] Romeo S. Sanyal A. andValenti L. (2020). Leveraging human genetics to identify potential new treatments for fatty liver disease. Cell Metab. 31, 35–45. 10.1016/j.cmet.2019.12.002 31914377

[B77] Sakurai Y. Kubota N. Yamauchi T. andKadowaki T. (2021). Role of insulin resistance in MAFLD. Int. J. Mol. Sci. 22, 4156. 10.3390/ijms22084156 33923817 PMC8072900

[B78] Salari N. Darvishi N. Mansouri K. Ghasemi H. Hosseinian-Far M. Darvishi F. (2021). Association between PNPLA3 rs738409 polymorphism and nonalcoholic fatty liver disease: a systematic review and meta-analysis. BMC Endocr. Disord. 21, 125. 10.1186/s12902-021-00789-4 34147109 PMC8214766

[B79] Sangro P. De La Torre Aláez M. Sangro B. andD’avola D. (2023). Metabolic dysfunction–associated fatty liver disease (MAFLD): an update of the recent advances in pharmacological treatment. J. Physiology Biochem. 2023. 10.1007/s13105-023-00954-4 PMC10635944 36976456

[B80] Scharner J. Aznarez I. (2021). Clinical applications of single-stranded oligonucleotides: current landscape of approved and in-development therapeutics. Mol. Ther. 29, 540–554. 10.1016/j.ymthe.2020.12.022 33359792 PMC7854307

[B81] Shang L. Mashek D. G. (2020). The underpinnings of PNPLA3-mediated fatty liver emerge. Hepatol. Baltim. Md 71, 375–377. 10.1002/hep.30888 31378994

[B82] Sharma D. Mandal P. (2022). NAFLD: genetics and its clinical implications. Clin. Res. Hepatol. Gastroenterol. 46, 102003. 10.1016/j.clinre.2022.102003 35963605

[B83] Stefano J. T. Duarte S. M. B. Ribeiro Leite Altikes R. G. andOliveira C. P. (2023). Non-pharmacological management options for MAFLD: a practical guide. Ther. Adv. Endocrinol. Metab. 14, 20420188231160394. 10.1177/20420188231160394 36968655 PMC10031614

[B84] Stender S. Loomba R. (2020). PNPLA3 genotype and risk of liver and all-cause mortality. Hepatology 71, 777–779. 10.1002/hep.31113 31954067 PMC7979358

[B85] Su W. Wu S. Yang Y. Guo Y. Zhang H. Su J. (2022). Phosphorylation of 17β-hydroxysteroid dehydrogenase 13 at serine 33 attenuates nonalcoholic fatty liver disease in mice. Nat. Commun. 13, 6577. 10.1038/s41467-022-34299-1 36323699 PMC9630536

[B86] Sveinbjornsson G. Ulfarsson M. O. Thorolfsdottir R. B. Jonsson B. A. Einarsson E. Gunnlaugsson G. (2022). Multiomics study of nonalcoholic fatty liver disease. Nat. Genet. 54, 1652–1663. 10.1038/s41588-022-01199-5 36280732 PMC9649432

[B87] Tang S. Zhang J. Mei T. T. Zhang W. Y. Zheng S. J. andYu H. B. (2021). Association of HSD17B13 rs72613567: tA allelic variant with liver disease: review and meta-analysis. BMC Gastroenterol. 21, 490. 10.1186/s12876-021-02067-y 34930143 PMC8686634

[B88] Teo K. Abeysekera K. W. M. Adams L. Aigner E. Anstee Q. M. Banales J. M. (2021). rs641738C>T near MBOAT7 is associated with liver fat, ALT and fibrosis in NAFLD: A meta-analysis. J. Hepatology 74, 20–30. 10.1016/j.jhep.2020.08.027 PMC7755037 32882372

[B89] Thangapandi V. R. Knittelfelder O. Brosch M. Patsenker E. Vvedenskaya O. Buch S. (2021). Loss of hepatic Mboat7 leads to liver fibrosis. Gut 70, 940–950. 10.1136/gutjnl-2020-320853 32591434 PMC8040158

[B90] Tilson S. G. Morell C. M. Lenaerts A. S. Park S. B. Hu Z. Jenkins B. (2021). Modeling PNPLA3-associated NAFLD using human-induced pluripotent stem cells. Hepatology 74, 2998–3017. 10.1002/hep.32063 34288010 PMC11497257

[B91] Ting Y.-W. Kong A. S.-Y. Zain S. M. Chan W.-K. Tan H.-L. Mohamed Z. (2021). Loss-of-function HSD17B13 variants, non-alcoholic steatohepatitis and adverse liver outcomes: results from a multi-ethnic asian cohort. Clin. Mol. Hepatology 27, 486–498. 10.3350/cmh.2020.0162 PMC8273635 33618508

[B92] Trepo E. Romeo S. Zucman-Rossi J. andNahon P. (2016). PNPLA3 gene in liver diseases. J. Hepatol. 65, 399–412. 10.1016/j.jhep.2016.03.011 27038645

[B93] Tsai E. Lee T. P. (2018). Diagnosis and evaluation of nonalcoholic fatty liver disease/nonalcoholic steatohepatitis, including noninvasive biomarkers and transient elastography. Clin. Liver Dis. 22, 73–92. 10.1016/j.cld.2017.08.004 29128062

[B94] Varadharajan V. Massey W. J. andBrown J. M. (2022). Membrane-bound O-acyltransferase 7 (MBOAT7)-driven phosphatidylinositol remodeling in advanced liver disease. J. Lipid Res. 63, 100234. 10.1016/j.jlr.2022.100234 35636492 PMC9240865

[B95] Vujkovic M. Ramdas S. Lorenz K. M. Guo X. Darlay R. Cordell H. J. (2022). A multiancestry genome-wide association study of unexplained chronic ALT elevation as a proxy for nonalcoholic fatty liver disease with histological and radiological validation. Nat. Genet. 54, 761–771. 10.1038/s41588-022-01078-z 35654975 PMC10024253

[B96] Wang J. Conti D. V. Bogumil D. Sheng X. Noureddin M. Wilkens L. R. (2021). Association of genetic risk score with NAFLD in an ethnically diverse cohort. Hepatol. Commun. 5, 1689–1703. 10.1002/hep4.1751 34558842 PMC8485887

[B97] Wang M. Li L. Xu Y. Du J. andLing C. (2022). Roles of hepatic stellate cells in NAFLD: from the perspective of inflammation and fibrosis. Front. Pharmacol. 13, 958428. 10.3389/fphar.2022.958428 36313291 PMC9606692

[B98] Wang Y. Kory N. Basuray S. Cohen J. C. andHobbs H. H. (2019). PNPLA3, CGI-58, and inhibition of hepatic triglyceride hydrolysis in mice. Hepatology 69, 2427–2441. 10.1002/hep.30583 30802989 PMC6563103

[B99] Wei Z. Huang Z. Song Z. Zhao W. Zhao D. Tan Y. (2023). Metabolic dysfunction-associated fatty liver disease and incident heart failure risk: the kailuan cohort study. Diabetology Metabolic Syndrome 15, 137. 10.1186/s13098-023-01102-0 37355613 PMC10290295

[B100] Xia M. Zeng H. Wang S. Tang H. andGao X. (2021). Insights into contribution of genetic variants towards the susceptibility of MAFLD revealed by the NMR-based lipoprotein profiling. J. Hepatol. 74, 974–977. 10.1016/j.jhep.2020.10.019 33340578

[B101] Xiang H. Wu Z. Wang J. andWu T. (2021). Research progress, challenges and perspectives on PNPLA3 and its variants in Liver Diseases. J. Cancer 12, 5929–5937. 10.7150/jca.57951 34476007 PMC8408107

[B102] Xu X. Poulsen K. L. Wu L. Liu S. Miyata T. Song Q. (2022). Targeted therapeutics and novel signaling pathways in non-alcohol-associated fatty liver/steatohepatitis (NAFL/NASH). Signal Transduct. Target Ther. 7, 287. 10.1038/s41392-022-01119-3 35963848 PMC9376100

[B103] Xue W. Y. Zhang L. Liu C. M. Gao Y. Li S. J. Huai Z. Y. (2022). Research progress on the relationship between TM6SF2 rs58542926 polymorphism and non-alcoholic fatty liver disease. Expert Rev. Gastroenterol. Hepatol. 16, 97–107. 10.1080/17474124.2022.2032661 35057689

[B104] Yang A. Zhu X. Zhang L. Zhang Y. Zhang D. Jin M. (2022). Non-invasive evaluation of NAFLD and the contribution of genes: an MRI-PDFF-based cross-sectional study. Hepatol. Int. 16, 1035–1051. 10.1007/s12072-022-10355-2 35829866

[B105] Yu L. Li Y. Grise A. andWang H. (2020). CGI-58: versatile regulator of intracellular lipid droplet homeostasis. Adv. Exp. Med. Biol. 1276, 197–222. 10.1007/978-981-15-6082-8_13 32705602 PMC8063591

[B106] Yuan F. Gu Z. Bi Y. Yuan R. Niu W. Ren D. (2022). The association between rs1260326 with the risk of NAFLD and the mediation effect of triglyceride on NAFLD in the elderly Chinese Han population. Aging (Albany NY) 14, 2736–2747. 10.18632/aging.203970 35333773 PMC9004570

[B107] Yuan S. Liu H. Yuan D. Xu J. Chen Y. Xu X. (2020). PNPLA3 I148M mediates the regulatory effect of NF-kB on inflammation in PA-treated HepG2 cells. J. Cell Mol. Med. 24, 1541–1552. 10.1111/jcmm.14839 31793207 PMC6991629

[B108] Zhang H. B. Su W. Xu H. Zhang X. Y. andGuan Y. F. (2021). HSD17B13: A potential therapeutic target for NAFLD. Front. Mol. Biosci. 8, 824776. 10.3389/fmolb.2021.824776 35071330 PMC8776652

[B109] Zhang Q. H. Yin R. X. Chen W. X. Cao X. L. andWu J. Z. (2019). TRIB1 and TRPS1 variants, G × G and G × E interactions on serum lipid levels, the risk of coronary heart disease and ischemic stroke. Sci. Rep. 9, 2376. 10.1038/s41598-019-38765-7 30787327 PMC6382757

[B110] Zhang X. Liu S. Dong Q. Xin Y. andXuan S. (2018). The genetics of clinical liver diseases: insight into the TM6SF2 E167K variant. J. Clin. Transl. Hepatol. 6, 326–331. 10.14218/JCTH.2018.00022 30271746 PMC6160302

[B111] Zhang X. Zhang B. Zhang C. Sun G. andSun X. (2021). Current progress in delineating the roles of pseudokinase TRIB1 in controlling human diseases. J. Cancer 12, 6012–6020. 10.7150/jca.51627 34539875 PMC8425202

[B112] Zhou T. Li H. Zhong H. Zhong Z. andLin S. (2020). Association of apoE gene polymorphisms with lipid metabolism in renal diseases. Afr. Health Sci. 20, 1368–1381. 10.4314/ahs.v20i3.43 33402986 PMC7751546

[B113] Zhu X. Xia M. andGao X. (2022). Update on genetics and epigenetics in metabolic associated fatty liver disease. Ther. Adv. Endocrinol. Metab. 13, 20420188221132138. 10.1177/20420188221132138 36325500 PMC9619279

